#  An Investigation into the Roles of Theory of Mind, Emotion Regulation, and Attachment Styles in Predicting the Traits of Borderline Personality Disorder

**Published:** 2016-10

**Authors:** Hamed Ghiasi, Abolalfazl Mohammadi, Pouria Zarrinfar

**Affiliations:** 1 PhD Student in Clinical Psychology, Shahid Beheshti University of Medical Sciences, Tehran, Iran.; 2 Assistant Professor of Clinical Psychology, Department of Psychiatry, Tehran University of Medical Sciences, Roozbeh Hospital,Tehran, Iran.; 3 MA in Clinical Psychology, Islamic Azad University, Science and Research Branch, Tehran, Iran.

**Keywords:** *Attachment Styles*, *Borderline Personality Disorder*, *Emotion Regulation*, *Theory of Mind*

## Abstract

**Objective:** Borderline personality disorder is one of the most complex and prevalent personality disorders. Many variables have so far been studied in relation to this disorder. This study aimed to investigate the role of emotion regulation, attachment styles, and theory of mind in predicting the traits of borderline personality disorder.

**Method:** In this study, 85 patients with borderline personality disorder were selected using convenience sampling method. To measure the desired variables, the questionnaires of Gross emotion regulation, Collins and Read attachment styles, and Baron Cohen's Reading Mind from Eyes Test were applied. The data were analyzed using multivariate stepwise regression technique.

**Results:** Emotion regulation, attachment styles, and theory of mind predicted 41.2% of the variance criterion altogether; among which, the shares of emotion regulation, attachment styles and theory of mind to the distribution of the traits of borderline personality disorder were 27.5%, 9.8%, and 3.9%, respectively.‎‎

**Conclusion**: The results of the study revealed that emotion regulation, attachment styles, and theory of mind are important variables in predicting the traits of borderline personality disorder and that these variables can be well applied for both the treatment and identification of this disorder.

Personality traits are sustainable patterns of the perception and thought about the self and environment that are found in a broad zone of social and personal context. When personality traits are non-flexible and non-adaptive and lead to the degradation of the performance and mental anguish, they make up personality disorder (1).

The maximal volume of studies on personality disorders is allocated to the borderline personality disorder (2-4). Borderline personality disorder is one of the most complex and serious mental disorders whose characteristics are persistent problems in regulating emotions, controlling impulses and instability in interpersonal relationships and self-image (1).

Many variables have been studied so far to identify borderline personality disorder including emotion regulation (5-7), attachment (8-11) and theory of mind (12-14).

Emotional dysregulation (ED) is known as one of the main signs in patients with borderline personality disorder (15, 16). Many studies have found that many borderline personality traits (such as self-harm, emotional instability, impulsivity, etc.) are derived from emotional dysregulation (5, 13). Studies also revealed that people with this disorder have problems to identify, distinguish and integrate their own emotions with those of others (5).

The concept of emotion regulation refers to the implicit and explicit efforts to increase positive modes and states and decrease the negative ones (17). The term emotion dysregulation pertains to the defect in the capacity of regulating emotions, especially when it is used in the case of borderline personality disorder; in this case, emotions become out of control, quickly change, and are expressed extremely and unmodified (18). Yen et al. (7) found that low ability to control emotions is associated with many signs of borderline personality disorder. Emotion regulation involves all external and internal processes responsible for monitoring, evaluating and modifying emotional reactions, especially its severe and transient states, to achieve the individual’s objectives that can be done consciously or unconsciously (19).

In addition, a number of symptoms of borderline personality disorder allocate to disorder in interpersonal relationships (20). In general, it may be argued that success in interpersonal relationships in social contexts requires the ability to take into account intentions, emotions, and opinions of others for decision making (21). Such ability refers to the theory of mind (22), mentalization (23) and empathy (24). Some theorists have used the words ‘empathy and theory of mind’ interchangeably (25, 26). However, other researchers made a distinction between empathy components of cognitive and emotional, and the theory of mind to cognitive empathy (14).

Theory of mind (ToM) implies the ability of documents in mental states to oneself and others in an attempt to understand and explain a behavior. According to the results of a study (14), patients with borderline personality disorder had deficits in the theory of mind. Psychological conditions involve a range of desires, intentions, beliefs, emotions and perceptions. Any failure to understand others’ desires and intentions leads to deficits of empathy and the reception of others’ opinions and cognitive distortions (23).

Other researchers and theorists have tried to explain the main traits and characteristics of borderline personality disorder based on attachment theory (8-10, 27, 28). Attachment theory has provided a conceptual framework and methodology to understand and diagnose mental representations (internal models) inconsistent with self and others that are an inseparable part of the development and stability for borderline personality disorder (29). According to the attachment theory, expressing emotion to the caregiver leads to the development of secure or insecure attachment (30). Secure attachment is related to the individuals’ ability in association with others and compatibility with emotional or stressful issues (31). If people have a caregiver who is stable in emotional capabilities, they are likely to achieve secure attachment and can effectively face negative events in life. However, if they do not have such a caregiver, they will develop insecure attachment and have less ability to emotional adaption (30). Levy (32) pointed out that the rate of secure attachment in patients with borderline personality disorder is at very low level compared to normal subjects and has been reported to be at the range of 0% to 30% and 6% to 8% on average.

Despite the prevalence of 1.6% to 5.9% of borderline personality disorder in the general population, prevalence of 20% in the clinical population (1) and the high risks and costs of this disorder for the community, to date no study has investigated the role of theory of mind, emotion regulation and attachment styles in Iran to predict the traits of borderline personality disorder in a clinical sample. 

Therefore, this study aimed to predict the traits of borderline personality disorder based on the theory of mind, emotion regulation and attachment styles in a clinical sample.

## Materials and Method

This was a field study with a non-experimental, descriptive, correlational and multivariate stepwise regression method. The study population consisted of patients aged 18- 55 yrs., with borderline personality disorder who referred to Roozbeh hospital and Rahaneshan Aria, Rahavard and Valaesh clinics. The sampling method was objective and convenience. The sample size had to be at least 10 times of the independent variables based on the statistical method of the study (multiple regressions) (33). Because the theory of mind, emotion regulation and attachment styles have one, two and three levels respectively, .85 cases of patients referring to Roozbeh hospital and Valaesh clinic were selected as the study sample.

The study inclusion criteria were as follows: Holding at least the fifth level of education in primary school, the diagnosis of borderline personality disorder by a psychiatrist or clinical psychologist, and the age range of 18 and 55 years. Exclusion criteria consisted of individuals who did not have any of the above criteria and the related physical diseases or psychosis.

***Measures***

***Adult Eye Test to Evaluate Theory of Mind:*** To assess the theory of mind, the revised version of Baron Cohen's Reading Mind from Eyes Test (RMET)     (25) was used. Eye test is an assessment instrument for facial expressions made to evaluate people in recalling the emotional symptoms. The test consists of 36 photographs of facial expressions which only show the area of the eyes. The participants are asked to select the most appropriate word out of four words that can better explain thoughts and feelings of the owner of photography and address his/her beliefs and feelings. For scoring, each correct response received a score between zero and 36. To evaluate the psychometric properties of this test, Mahmood-Alilo and colleagues(34), carried out a preliminary study with a sample of 100 through translating the English version by professors of English. The obtained validity and internal consistency of the test (Cronbach's alpha) was 73/0. Also, NejatiSafa and colleagues (35) who addressed the relationship between mindfulness and reading the mind from the eye, used this test and found the test to have adequate and appropriate validity.

***Collins and Read Revised Adult Attachment Scale (*** 36 ***):*** This scale contains self-assessment of skills of creating relationships and self-description of the way attachment relationships are formed to close attachment figures. It consists of 18 articles measured by marking on a five- point scale (Likert), with score of one (not corresponding to my traits) to five (completely corresponding to my traits).Through analyzing the factors, 3 subscales with 6 articles were determined which were as follows: Secure, avoidant, and ambivalent attachments; each of which is scored from zero to four. Therefore, the range of changes for each scale is from zero to 24. Pakdaman (37) reported the rate of Cronbach's Alpha to be 0.81, 0.78, and 0.85 for secure, avoidant and ambivalent subscales, respectively. In the study by Pakdaman, the test - retest reliability was obtained to be 0.95 for this scale.

***Emotion Regulation Questionnaire (ERQ):*** This 10-item questionnaire has two subscales of suppression and reappraisal. The items are ranked based on a 7-item scale. Gross and John (38) reported that the reliability and validity of the instrument was satisfactory. The alpha coefficient was obtained as 0.79 and 0.73 for reappraisal and suppression, respectively; and the 3-month retest reliability was obtained to be 0.69 for both subscales. This instrument has also been used for Iranian samples (39). The psychometric properties of the instrument were preliminarily evaluated for this study. In the preliminary study performed on 41 students (with the mean age (standard deviation) of 22.75 (2.30)), the internal consistency was obtained to be 0.76 and 0.71 for reappraisal and suppression, respectively. Moreover, the reliability of the retest was 0.67 and 0.64 for reappraisal and suppression, respectively for the Iranian version after one month correlation (n = 32).

***Schizotypal Trait Questionnaire-B Form (Borderline Personality Scale):*** Borderline personality scale is a part of Schizotypal Trait Questionnaire and borderline personality scale made by Claridge and Broks (40). Through adapting the edited version of the test with the criteria of the diagnostic and statistical manual, Mohammadzade, Goudarzi, Taghavi and Mollazade (41) introduced 24 articles which measured three factors of hopelessness, impulsivity and stress-related paranoia and dissociative symptoms on a Likert scale. Mohammadzade et al. (41) reported that the reliability of the questionnaire was about 0.84, 0.53, 0.72, and 0.50 for the whole borderline personality scales and subscales of hopelessness, impulsivity, and stress-related paranoia and dissociative symptoms, respectively, with the alpha coefficient of 0.77, 0.64, 0.58, and 0.57, respectively.

***Structured Clinical Interview of DSM-IV-TR for Axis II:*** This instrument is a semi-structured diagnostic interview formulated by Gibbon, Spitzer and First (42) to assess 10 axis II personality disorders. By this instrument, axis II disorders can be specified into two forms of classification (the presence or absence of personality disorder) considered in the study or dimensions (considering the criteria for personality disorder). With regards to the reliability of SCID-II, some studies found a high reliability of the test. Kappa coefficient varied from 0.24 for compulsive personality disorder to 0.74 for histrionic personality disorder (with total Kappa index of 0.53) and the consensus among the assessors was significantly reported less than the total Kappa of 0.38 (42). The content validity of the translated version of the test in Iran was confirmed in the study by Bakhtiari (43) and the reliability coefficient of the test was obtained to be 0.87 using test- retest method in one week interval.

***Demographic Questionnaire:*** This questionnaire included demographic questions, history of patients and previous treatments for individuals.

***Implementation***

This study was conducted on patients with borderline personality disorder who referred to Roozbeh hospital and Valayesh clinic. The study sample was selected using convenience sampling method and patients with borderline personality disorder were selected based on the diagnosis of a clinical psychiatrist or a psychologist using a semi-structured interview for axis II disorders.

After determining the sample and obtaining oral and written consent and discussing the aims of the study, a clinical psychologist conducted the questionnaires and instruments. The questionnaires and instruments were administered in random so that the effect of fatigue and other related variables could be prevented on the study results. Data entered into the computer, described using SPSS software, and analyzed running multivariate regression. The obtained results were discussed according to the study literature.

## Results

The study sample consisted of 85 patients with borderline personality disorder. The minimum age of the sample was 18, while the maximum age was 46; their age range was 28 years. In terms of sex, out of 85 individuals, 24 were male and 61 were female. The mean (and standard deviation) of borderline personality traits was 14.36 (3.37), theory of mind: 27.76 (3.54), emotion regulation (Suppression): 20.92 (2.583), emotion regulation (Reappraisal): 18.77 (5.19), secure attachment styles: 9.07 (5.78), avoidance attachment: 12.91 (24.05) and ambivalent attachment was 13.4 (4.17), respectively. The correlation coefficient and significance level of the above-mentioned are listed in Table 1.

To examine the multiple linear correlations, we used matrix of bivariate correlation (Table 1) and the index of tolerance and variance inflation factor. Given that multiple linear correlations occur at above 0.8 and considering the correlation matrix, no such high correlation was found between the predictor variables. The tolerance index of all the variables were at least 0.912 and utmost 1, and the variance inflation factor was 1 at the minimum and 1.097 at the maximum, indicating that there was not b any multiple linearity, and predictor variables were relatively independent of each other. Stepwise regression analysis revealed a significant relationship between the predictor variables and criterion. 

**Table1:** Correlation Coefficients Between Borderline Traits, Theory of Mind, Emotion Regulation (Reappraisal, Suppression) and Attachment Styles, and Their Levels of Significance

	1	2	3	4	5	6	7
Borderline traits		0.003	0.001	0.001	0.025	0.007	0.054
Theory of mind	0.296		0.040	0.287	0.491	0.027	0.318
Reappraisal	-0.39	-0.19		0.369	0.402	0.459	0.269
Suppression	0.357	-0.062	-0.037		0.398	0.302	0.094
Ambivalent attachment	0.214	-0.002	0.027	0.028		0.102	0.005
Secure attachment	-0.26	-0.209	-0.011	-0.058	-0.139		0.008
Avoidance attachment	0.17	0.052	-0.068	0.144	0.278	-0.36	

**Table2:** Summary of the Result of Regression Analysis of Emotion Regulation (Reappraisal, Suppression), Attachment Styles and Theory of Mind in Predicting Borderline Personality Traits

**Model**	**R**	**R** ^2^	**Adjusted ** **R** ^2^	**Std. ** **error**	**Statistical ** **changes**				
					**R** ^2^ **changes**	**F ** **changes**	**df1**	**df2**	**Level of ** **significance**
1	0.396^a^	0.157	0.147	3.116	0.157	15.434	1	83	0.001
2	0.524^b^	0.247	0.257	2.908	0.118	13.299	1	82	0.001
3	0.581^c^	0.338	0.313	2.795	0.063	7.765	1	81	0.007
4	0.614^d^	0.377	0.346	2.727	0.039	5.070	1	80	0.027
5	0.642^e^	0.412	0.357	2.666	0.035	4.693	1	79	0.033

f. Independent variable: Borderline personality traits

a . Predictor: Reappraisal

b . Predictor: Reappraisal, suppression

c . Predictor: Reappraisal, suppression, secure attachment

d . Predictor: Reappraisal, suppression, secure attachment, theory of mind

e . Predictor: reappraisals, suppression, secure attachment, theory of mind, ambivalent attachment

**Table3:** T-Tests on the Weight of Emotion Regulation (Reappraisal, Suppression), Attachment Styles and Theory of Mind in Predicting Borderline Personality Traits

**Model**	**Variables**	**standardized coefficients**		**Non standardized coefficients**		**Level of significance**
		**b**	**Standard error**	**Beta**	**t**	
1	Reappraisal	-2.057	0.065	-0.396	-3.929	0.001
2	Reappraisal	-0.249	0.061	-0.383	-4.072	0.001
	Suppression	0.448	0.123	0.343	3.647	0.001
3	Reappraisal	-0.251	0.059	-0.387	-4.274	0.001
	Suppression	0.429	0.118	0.329	3.628	0.001
	Secure attachment	-0.147	0.053	-0.253	-2.787	0.007
4	Reappraisal	-0.224	0.058	-0.346	-3.837	0.001
	Suppression	0.451	0.116	0.346	3.896	0.001
	Secure attachment	-0.121	0.053	-0.207	-2.289	0.025
	Theory of mind	0.198	0.088	0.208	2.252	0.027
5	Reappraisal	-0.227	0.057	-0.350	-3.969	0.001
	Suppression	0.447	0.113	0.342	3.941	0.001
	Secure attachment	-.0.105	0.052	-0.180	-2.015	0.047
	Theory of mind	0.203	0.086	0.213	2.359	0.021
	Ambivalent attachment	0.153	0.070	0.189	2.166	0.033

As demonstrated in Table 2, two levels of emotion regulation (i.e., the variables of reappraisal in the first step and suppression in the second step) were entered into the model; reappraisal could predict 15.7% of the dispersion of the borderline personality traits as the best predictor variable, while suppression predicted 11.8% of the dispersion of the borderline personality traits in the second step. 

In this model, two styles of secure and ambivalent attachment were entered into the model. Secure attachment style in the third step (R2 changed to 0.063) and ambivalent attachment style in the fifth step (R2 changed to 0.035) were entered into the model and predicted 9.8 % of the variance in borderline personality traits. 

Moreover, 3.9% of the variance criterion was predictable by the theory of mind, which was entered into the model in the fourth step (R2 changed to 0.039).

Thus, emotion regulation, attachment styles and theory of mind predicted 41.2% of the variance criterion altogether. Among them, emotion regulation, attachment styles and theory of mind predicted 27.5%, 9.8% and 3.9% of the dispersion of borderline personality traits, respectively.

Considering the coefficients of b and standard error, (which are not standardized coefficients) and standardized beta coefficients as well as the outputs of t-tests, the weight of each of the variables in the regression was examined at each step Table 3. Given the t-tests and their significant levels, the share of each variable in predicting the criterion variable could be evaluated. Reappraisal, suppression, secure attachment, theory of mind and ambivalent attachment were significant at the alpha level of 0.05 for predicting borderline personality traits.

## Discussion

This study aimed to predict the traits of borderline personality disorder based on theory of mind, emotion regulation and attachment styles in a clinical sample. Emotion regulation (reappraisal) was entered into the model at the first step. In line with previous results (5-7, 34 and 44), the strong, significant and reverse relationship of this variable with the criterion variable (traits of borderline personality) confirms that individuals with borderline personality disorder have a deficit in emotion regulation. Emotional dysregulation (ED) is known as one of the main symptoms in patients with borderline personality disorder (15, 16). Studies have found that many traits of borderline personality (such as self-harm, emotional instability, impulsivity, etc.) are derived from emotional dysregulation (5, 13). In explaining these results, Gross (45) stated that the reappraisal of the strategy of emotion regulation is so effective that it reduces experience and tools of emotional behavioral tool. Since emotion regulation plays an essential role in interpersonal relationship, conveying emotions to others, and in creating, maintain and cutting relationships with others (46), weakness in the reappraisal of emotions can lead to the development and maintenance of emotion and personality disorders (45) such as borderline personality disorder.

The results of this study revealed a significant and direct relationship between suppression and traits of borderline personality disorder. The results are consistent with the results of previous studies (47-49). In explaining these results, Gross and John (38) stated that suppression is a type of strategy for problematic emotion regulation, which is associated with the high levels of negative emotions and interpersonal problems. This emotion strategy reduces the behavioral tool of emotion. However, it is unable to decrease the experience of emotion and it suppresses the physiological responses in person and increases them in the community. Also, this strategy causes to remain negative thoughts in the mind more and even increase the rate of thoughts (50). In their study, Gross and John (38) found that individuals who used suppression strategy, experienced less positive and more negative emotions. In addition, using suppression has a relationship with worse interpersonal performance. Suppression strategy is applied for unpleasant emotion regulation that results in more discomfort and inefficiency. Suppression is a strategy based on response and actively prevents the emotion expression by the individual (19). This inhibition leads to invade uncomfortable thoughts to the individual at a higher level in the next times and to threaten mental health (51). Therefore, many signs and symptoms in patients with borderline personality disorder such as impulsivity and interpersonal problems can be considered to be caused by emotion suppression.

In the third step, secure attachment had necessary criteria for inclusion in the model. After the variables of emotion regulation (reappraisal and suppression), this variable had the highest revers correlation with the criterion variable. These results are consistent with several studies (52-54), and not consistent with some others (55, 56). These contradictory results may be due to the samples were non-clinical. Moreover, the researchers proposed that borderline traits at the level of clinical semiotics might be related to the experience of serious communication problems; and in fact, borderline personality disorder is explained by “fluctuation” in the attachment greater than common forms of insecure attachment. In other words, this hypothesis has proposed consistent attachment strategies as part of the insecure attachment styles (57).

After emotion regulation (reappraisal and suppression) and secure attachment, theory of mind enters into the model. The variable remains among the other variables and has the most relationship with the criteria variable after variables of emotion regulation. In this study, it has been found that patients with borderline personality disorder have a high ability for the theory of mind. The results are consistent with those of previous studies (12, 14 and 58). In explaining these results, Astington (59) suggested that aggressive and impulsive individuals are unable to receive others’ feelings, predict others’ behavior, and adjust their behavior based on the others’ behavior for processing certain information, and they are unable to empathize with others. People with borderline personality disorder reflect often inappropriate or extreme anger or suffer from problems in controlling their anger (1). In another explanation, Sharp et al. (13) recognizes emotion dysregulation as the cause of ultra-reading the mind in patients with borderline personality disorder because it has been revealed that the cause of interpersonal behavioral disorders in patients with borderline personality disorder is the disorder in emotion regulation. In examining the theory of mind in individuals with borderline personality disorder, it was found that emotion dysregulation causes ultra-reading of the mind or unusual and wrong reading of the mind.

Ambivalent and avoidant attachments are other variables that have a more significant relationship with the criterion variable. In regression analysis, ambivalent attachment was the last variable to be entered into the model, and avoidant attachment could not significantly predict the criterion variable. These results are consistent with the results of several studies (32, 52, 53, 56 and 60). The researchers and theorists stated various fundamental aspects of borderline personality disorder such as instability, difficult interpersonal relationships, feelings of emptiness, anger explosion, chronic fear of abandonment, inability to tolerate the feeling of loneliness, and the lack of stability, and they (8-11, 61) acknowledged the theory of attachment as both a pathological development and normal growth. Bowlby believed that attachment problems increase psychological vulnerability and help identify certain types of problems. He believed that insecure attachment in childhood leads to the lack of full formation of capacity for emotional relationship, and also a series of dysfunction in adulthood including marital problems, neurotic symptoms and personality disorders. Bowlby claimed that insecure attachment is at the core of syndrome of personality disorders (32). Maine (62) noted that the active internal model of patients with borderline personality disorder on self and attachment figure is multiple, fragmented and inconsistent. Evidence cited in longitudinal studies with regards to the relationship between the disorder and dissociative syndrome indicates that this disorder in children leads to a syndrome in adulthood, which is similar to borderline personality disorder (32).

## Limitations

This study had some restrictions. The study limitations are as follows: 1) Using convenience sampling method which made the generalization of the results difficult; 2) Lack of control of patients’ mood within the performance of the questionnaires and other variables that may influence the results; 3) Lack of distinction between inpatient and outpatient samples that may have influenced the results of the study; 4) Lack of a direct study on the relationship between theory of mind and traits of borderline personality disorder in patients with the disorder in Iran, which made comparing the results of the study with those of others difficult; 5) No distinction of gender in the data analysis; 6) Lack of control on the type and dose of taking drugs by the patients. Addressing several studies, Russel (63) in his article "Is There Universal Recognition of Emotion from Facial Expression? A Review of the Cross-Cultural Studies", stated that recognition of emotion from facial expression is different in every culture. However, he mainly associates this difference with societies that have higher cultural relations and literacy level and isolated and illiterate societies. In fact, Russell's study indicates that societies affected by Western culture represent a similar behavior in recognition of emotion from facial expression. Nevertheless, due to the lack of normalization of Baron Cohen's Reading Mind from Eyes Test and disagreement over penetration level of Western culture in Iran, the results of this study should be interpreted with caution.

## Conclusion

The results show that emotion regulation, attachment styles and theory of mind, are important variables in predicting borderline personality disorder traits and can take advantage of these variables both to treat and to more identify the disorder.
